# A case report on the co-presentation of pituitary abscess and POEMS-like syndrome

**DOI:** 10.1097/MD.0000000000050508

**Published:** 2026-09-11

**Authors:** Xiangjin Hu, Heng Wu, Li Fang

**Affiliations:** a Department of Hepatology and Infectious Diseases, Zhejiang University School of Medicine Sir Run Run Shaw Hospital, Hangzhou, China.

**Keywords:** Diabetes insipidus, Hormone replacement therapy, Panhypopituitarism, Pituitary abscess, POEMS syndrome, Transsphenoidal surgery

## Abstract

**Rationale:**

POEMS syndrome is a rare paraneoplastic syndrome, manifesting with progressive distal polyneuropathy, organomegaly, endocrinopathy, monoclonal protein, and skin changes, which has multisystem manifestations and chronic cytokine overproduction. Pituitary abscess is also a rare life-threatening disease that could lead to panhypopituitarism. We report a case of pituitary abscess with high suspicion of POEMS syndrome, which to our knowledge has not been reported previously.

**Patient concerns:**

The co-presentation of pituitary abscess and POEMS-like syndrome is rare and its clinical manifestations are complex, making it easy to miss diagnosis and misdiagnosis. Long-term disease management and follow-up is crucial under this condition.

**Diagnosis:**

The patient was admitted to outside hospital due to headache, impaired consciousness, and fever since April 2024. Initial test results suggested neuroinfection. Endocrine abnormalities revealed diabetes insipidus and panhypopituitarism, including central hypocortisolism, hypogonadism, and hyperprolactinemia. Pituitary-enhanced MRI revealed abnormal pituitary signals on T1- and T2-weighted images, with peripheral rim enhancement noted after gadolinium injection. Further examination revealed lymphadenopathy, polyneuropathy, skin changes, monoclonal gammopathy (lamda chain, M-protein), ascites, meeting the diagnostic criteria for POEMS syndrome. Bone marrow core biopsy showed no malignancy. Despite the normal VEGF level, the patient was diagnosed with pituitary abscess and possibly POEMS syndrome due to other clinical presentations.

**Interventions:**

The patient underwent transsphenoidal resection of the pituitary gland lesions, antibiotic treatment and hormone replacement therapy with hydrocortisone and desmopressin.

**Outcomes:**

At 9 months of follow-up, the patient experienced recurrent fever, headache, and diabetes insipidus after discontinuation of hormone therapy.

**Lessons:**

The diagnosis of complex comorbidities is based on the combination of clinical manifestations and laboratory tests, multidisciplinary and comprehensive assessment are of great significance for this disease.

## 1. Introduction

Pituitary abscess is a rare, potentially life-threatening condition that may be misdiagnosed, accounting for <1% of all pituitary lesions.^[1]^ Clinical diagnosis is challenging due to the absence of specific symptoms and the similarity of imaging features to other pituitary lesions.^[2]^ The most common symptoms of pituitary abscess include headache, visual deficits, neurological symptoms, hypopituitarism, and diabetes insipidus, with fever or meningitis being rare. Endocrine abnormalities range from single hormone deficiencies to multiple deficiencies, or even panhypopituitarism.^[3]^ Notably, the presence of diabetes insipidus may suggest the possibility of a pituitary abscess, as its incidence in pituitary adenomas is <10%.^[4]^ Typical rim enhancement was observed on multimodal magnetic resonance imaging(MRI) after gadolinium injection,^[5]^ mainly manifested by signs of at least one sign of adjacent anatomical structure invasion, such as peripheral meningeal enhancement, pituitary stalk thickening, or paranasal sinus mucosal enhancement.^[6]^

POEMS syndrome is a rare paraneoplastic syndrome, represented by five major features of polyneuropathy, organomegaly, endocrinopathy, monoclonal gammopathy, and skin changes. The diagnosis of POEMS syndrome is based on the combination of clinical manifestations and laboratory tests.^[7]^ The multisystem involvement and rarity of POEMS syndrome lead to a strong possibility of missed diagnosis and misdiagnosis. Furthermore, comorbidities and atypical presentation can further complicate the diagnosis.

We report an unique case of a patient with pituitary abscess who presented with headache, impaired consciousness, fever and features of POEMS-like syndrome without elevated VEGF levels. Three months after transsphenoidal resection of the pituitary gland lesions, the patient developed recurrent fever, headache, and diabetes insipidus after hormone therapy was stopped. Besides, in February 2025, the patient developed ascites, a clinical feature that supported the diagnosis of POEMS syndrome, which was not found during the previous hospitalization. This case emphasizes the importance of comprehensive assessment and follow-up for complex comorbidities, while also reflecting the real-world challenges faced in diagnosing POEMS syndrome.

## 2. Case presentation

A 79-year-old male farmer with a history of skin pigmentation on both lower limbs was initially admitted to an outside hospital due to fever and severe headache lasting for 2 weeks. The patient experienced a transient episode of altered consciousness, followed by temporal disorientation and lethargy. Neurological examination revealed neck stiffness and a positive Brudzinski sign. Laboratory tests showed mild leukocytosis (leukocyte count: 9.5 × 10^9/L, neutrophils: 63%), elevated C-reactive protein (CRP: 19.68 mg/L), decreased serum chloride (94.4 mmol/L), and normal liver function. Cerebrospinal fluid (CSF) analysis revealed a markedly elevated leukocyte count (591 × 10^6/L, predominantly polymorphonuclear monocytes), with normal glucose and chloride levels. Autoimmune encephalitis-related antibodies were negative, while next-generation sequencing (NGS) of the CSF identified Epstein-Barr virus (EBV). Empirical treatment with meropenem was initiated for suspected bacterial meningitis. Head CT and contrast-enhanced MRI revealed no abnormalities. A follow-up CSF analysis showed an improvement in leukocyte count and protein levels; however, the patient’s condition did not improve. The patient developed high fever, nausea, vomiting, and worsening consciousness, and was subsequently transferred to our facility.

On arrival, physical examination revealed persistent headache, fever, lethargy, neck stiffness, and a positive Brudzinski sign. Given the clinical presentation, meningitis was strongly suspected, and empiric broad-spectrum antibiotic therapy with meropenem was continued. During hospitalization, serum autoantibodies including ANA, ANCA, tumor markers, and paraneoplastic antibodies were all negative. However, serum T-SPOT was positive, and serum EBV-DNA was significantly elevated (3.48 × 10^4 copies/mL). Cryptococcal capsular antigen tests in both serum and CSF were negative. Repeat CSF analysis showed increased protein concentration (1020 mg/L; normal < 450 mg/L), normal glucose (2.93 mmol/L, with concurrent serum glucose at 7.64 mmol/L), and adenosine deaminase of 2.0 IU/L. CSF Gene Xpert MTB/RIF, acid-fast bacilli stain, India ink stain, and Gram stain were all negative. No pathogens were isolated from blood cultures, but EBV was again identified in the CSF by NGS. Electroencephalography (EEG) revealed mild abnormalities. Because of limb weakness, electromyography (EMG) was performed, revealing absent responses from the right common peroneal, tibial, and superficial peroneal nerves, as well as reduced conduction velocity in the right sural, median, and ulnar nerves, consistent with peripheral neuropathy affecting both the upper and lower extremities. Visual field testing was not feasible because of the patient’s poor cooperation.

Although the patient’s CSF indicators improved, his clinical symptoms did not. Based on literature-reported common pathogens of bacterial meningitis,^[8,9]^ treatment was adjusted to ceftriaxone and vancomycin. Coincidentally, on the 7th day of hospitalization, the patient developed coronavirus disease 2019 (COVID-19), which led to recurrent high fever, simnotrelvir-ritonavir was then used for the treatment of COVID-19. The occurrence of COVID-19 initially affected judgment of the disease, but subsequent examinations helped us discover the patient’s comorbidities. Subsequently, serum M-protein, lamda chain were detected. The patient then developed frequent urination, and total and free prostate-specific antigen (PSA) levels were undetectable. Endocrine testing confirmed hypopituitarism (Table 1), combined the facts that monoclonal gammopathy characterized by M-protein and lamda chain, and peripheral neuropathy affecting both upper and lower limbs evidenced by electromyography, pituitary disease and POEMS syndrome were suspected.

**Table 1:** Endocrinologic laboratory data.

Test	Value	Normal range with units
Morning serum cortisol	1.45	6.7–22.6μg/dL
Morning adrenocorticotropic hormone	20	10–80ng/L
Luteinizing hormone	0.18	1.24–8.62IU/L
Follicle-stimulating hormone	0.56	1.27–19.26IU/L
Thyroid-stimulating hormone	1.54	0.35–4.94mIU/L
triiodothyronine	0.49	0.64–1.52ng/mL
insulin-like growth factor-1	40.9	54.8–185ng/mL
Testosterone	0.09	1.75–7.81μg/L
prolactin	25.53	2.64–13.13μg/L

**Figure 1.**
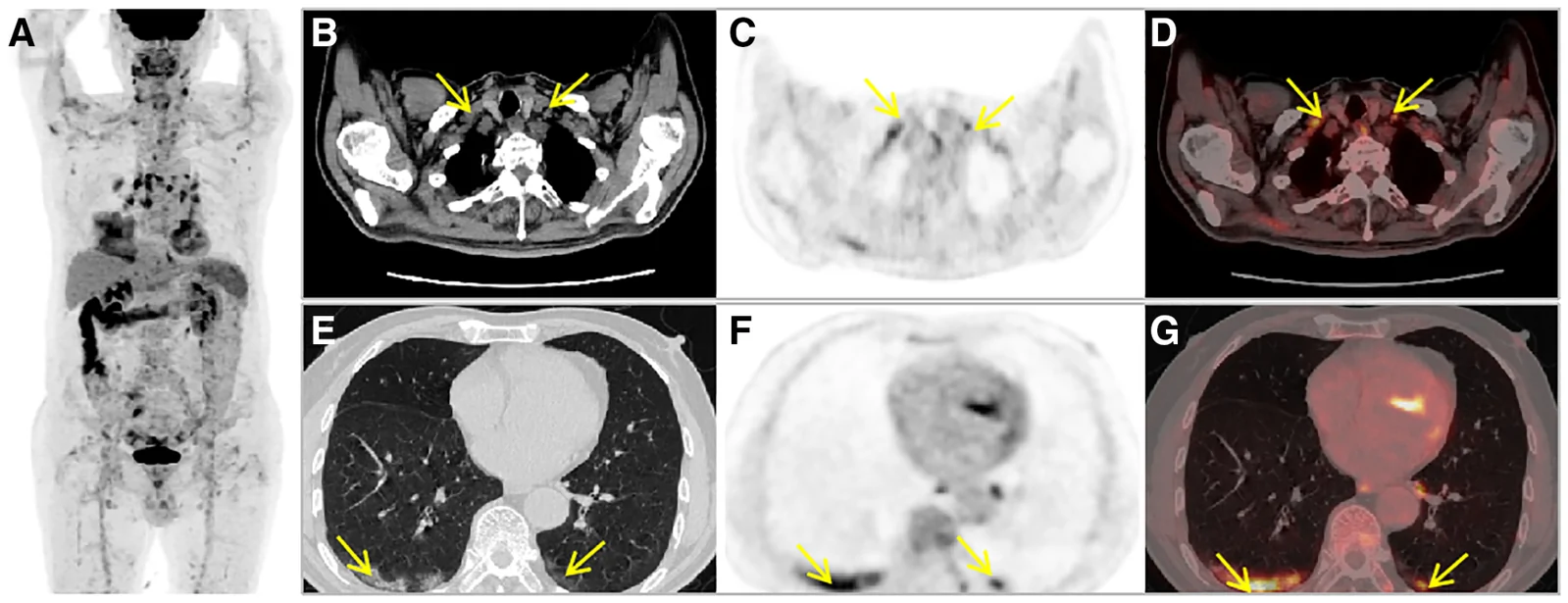
^18^F-FDG-PET/CT of the patient. (A) Maximum Intensity Projection (MIP) image of the chest, and upper and lower abdomen, respectively (left to the right) showed increased FDG uptake in the multiple lymph nodes (including cervical, thoracic, axillary, mediastinal and inguinal lymph nodes), lung, part of the colon and spleen. (B) Trans-axial CT images, (C) PET images, (D) Fused PET/CT images showed multiple abnormal foci of tracer uptake in the cervical lymph nodes. (E) Trans-axial CT images, (F) PET images, (G) Fused PET/CT images showed scattered patchy shadows in the lungs with elevated FDG (yellow arrow).

**Figure 2.**
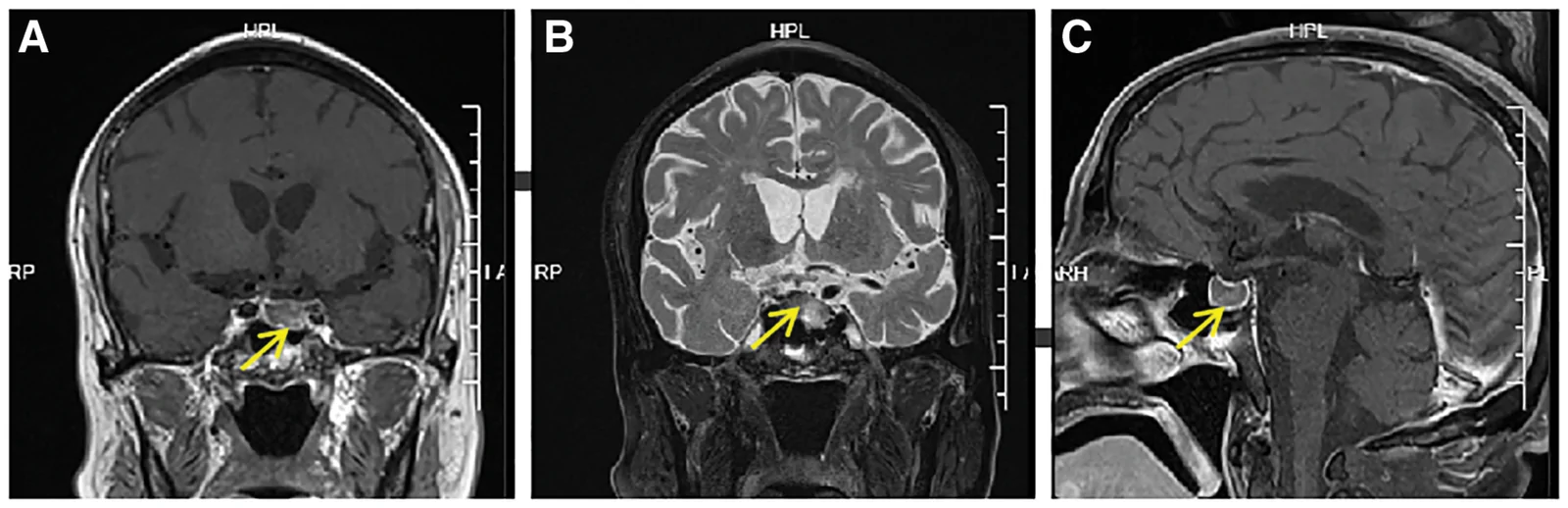
Enhanced MRI of the pituitary gland. (A) Coronal T1-weighted images, (B) Coronal T2-weighted images demonstrated that the pituitary stalk was not clearly displayed with mixed abnormal signal. (C) Sagittal T1-weighted images showed rim enhancement after gadolinium injection (highlighted by the arrow).

**Figure 3.**
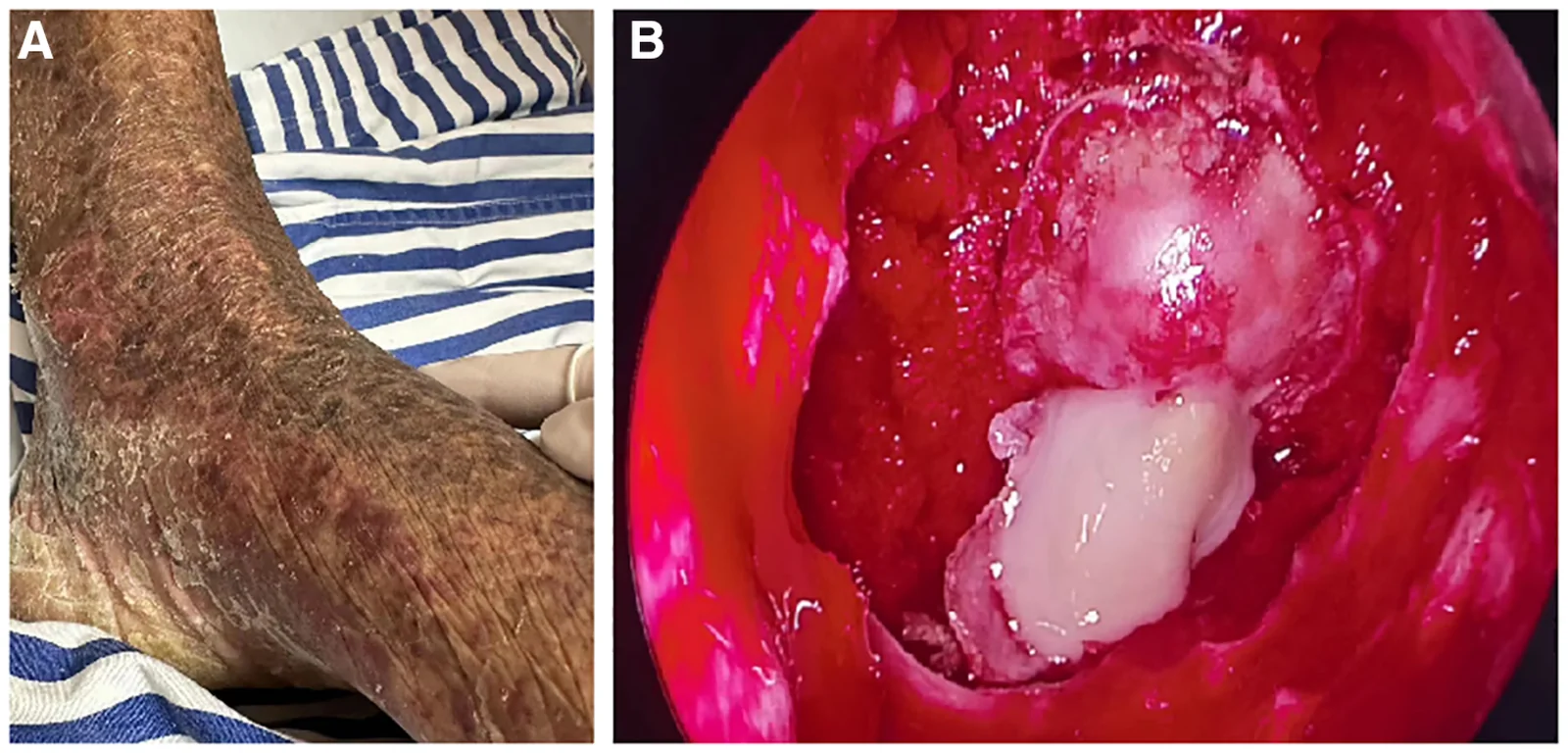
The image of the patient. (A) Image showed skin pigmentation of lower limbs. (B) Operative images showing yellowish pus of the pituitary gland.

Imaging examination, whole body ^18^F-fluorodeoxyglucose (FDG) positron emission tomography/computer tomography (PET/CT) scan and bone marrow were performed. PET/CT revealed increased FDG uptake in enlarged lymph nodes (SUV max, 11.4), including cervical, thoracic, axillary, mediastinal and inguinal lymph nodes (Fig. 1A–D), and bilateral pneumonia with FDG avidity (SUV max, 6.6) (Fig. 1E–G). There was no ^18^F-FDG uptake and osteosclerosis in the bone lesions. Bone marrow biopsy confirmed that the proportion of red blood cells and granulocytes were generally normal, and mature plasma cells constituted 1% of cells. Flow-cytometric examination of bone marrow showed no significant evidence of monoclonal plasma cells and other abnormal cells. VEGF showed normal levels. Brain CT and contrast-enhanced MRI scans from the referring hospital were reevaluated by a radiologist, who noted an enlarged pituitary gland with heterogeneous signal intensity and marked peripheral enhancement, raising suspicion for underlying pituitary pathology. The nasopharyngeal laryngoscopy findings were unremarkable. Subsequent contrast-enhanced pituitary MRI revealed an indistinct pituitary stalk, mixed signal intensities on both T1- and T2-weighted images, and peripheral rim enhancement following gadolinium administration, suggestive of differential diagnoses including pituitary adenoma with cystic degeneration, pituitary abscess, or hemorrhagic adenoma (Fig. 2). Based on MRI findings, serum and urine osmolality, and the clinical presentation, the diagnosis of diabetes insipidus and pituitary abscess was made. Given the multisystem involvement-diffuse lymphadenopathy, polyneuropathy, skin changes (Fig. 3A), monoclonal gammopathy (lambda chain, M-protein), and endocrine dysfunction-POEMS syndrome was suspected by a hematologist, despite the normal VEGF level.

**Figure 4.**
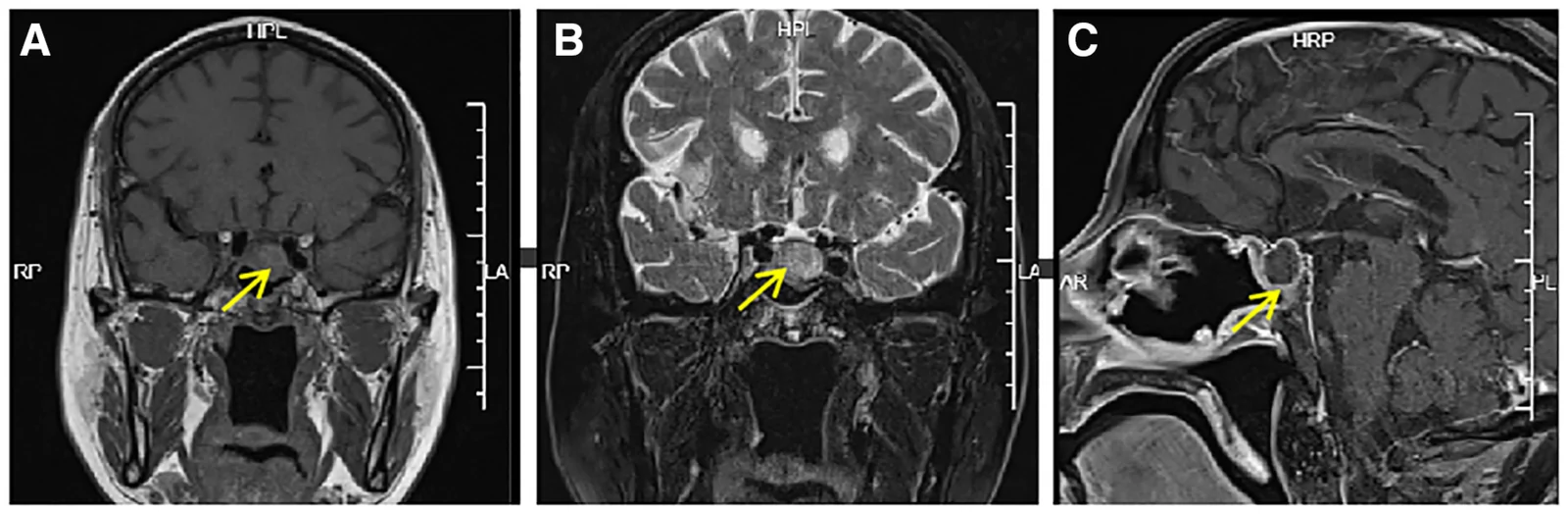
Postoperative pituitary-enhanced MRI. (A) Coronal T1-weighted images, (B) Coronal T2-weighted images showed mixed abnormal signals on the left side of the sella turcica, which broken through the sella turcica septum upward. (C) Sagittal T1-weighted images showed rim enhancement after gadolinium injection (highlighted by the arrow).

On hospital day 15, the patient’s fever, lethargy, and headache showed improvement. Hormone replacement therapy with hydrocortisone and desmopressin was initiated, along with vancomycin monotherapy. Considering current approaches to pituitary disorders, the patient underwent endoscopic endonasal transsphenoidal surgery for diagnostic and decompressive purposes. Intraoperatively, yellowish pus was observed within the pituitary gland (Fig. 3B). Specimens were collected for culture, NGS, and cytological examination. Cytology showed infiltration of lymphocytes and monocytes, but both culture and NGS were negative. Postoperatively, the patient continued on antibiotics for an additional 2 weeks (with a total antibiotic course of approximately 8 weeks), alongside ongoing hormone replacement therapy. The patient was discharged with instructions for an outpatient neurosurgical follow-up. The patient was lost to follow-up for 9 months, and later re-presented with recurrent fever, headache, and a recurrence of diabetes insipidus after discontinuing hormone therapy. Symptoms partially improved following intermittent antibiotic treatment at a local hospital. Additionally, in February 2025, the patient developed ascites, a clinical feature supportive of the POEMS syndrome diagnosis, which had not been previously evident. During the three-month postoperative period, follow-up MRI revealed a bulky pituitary gland with mixed signal intensities on T1- and T2-weighted sequences and persistent peripheral rim enhancement after gadolinium administration (Fig. 4). Persistently low cortisol levels necessitated continued hormone replacement therapy (Table 2). Comparison of serial imaging indicated progression of pituitary involvement relative to previous studies.

**Table 2:** The assessments of pituitary gland function 6 months after operation.

Test	Value	Normal range with units
Morning serum cortisol	1.01	6.7–22.6μg/dL
Morning adrenocorticotropic hormone	<1	10–80ng/L
Luteinizing hormone	0.26	1.24–8.62IU/L
Follicle-stimulating hormone	0.71	1.27–19.26IU/L
Thyroid-stimulating hormone	1.14	0.35–4.94mIU/L
triiodothyronine	0.44	0.64–1.52ng/mL
Testosterone	0.01	1.75–7.81μg/L
prolactin	21.84	2.64–13.13μg/L

## 3. Discussion

Pituitary abscess is a rare condition often associated with a poor prognosis and a high rate of misdiagnosis because of the nonspecific symptoms and imaging features. Its exact etiology and pathogen are unknown. Risk factors for pituitary abscess include immunosuppressive conditions such as diabetes mellitus, Cushing’s disease, and human immunodeficiency virus (HIV) infection.^[10]^ Gram-positive pathogens, such as Staphylococcus and Streptococcus species, are often reported in pituitary abscesses. However, almost half of the patients did not have pathogen isolation in pituitary infectious diseases,^[11–14]^ and half required permanent hormone replacement therapy.^[14]^ In a retrospective review of 84 primary pituitary abscess cases by Mallereau et al, a bacterial agent was identified in only 25% of cases, including Staphylococcus aureus, Streptococcus, Escherichia coli, and Pseudomonas aeruginosa. Gram-positive cocci accounted for over half of the identified pathogens, while Gram-negative bacilli were detected in 14.2% of cases.^[15]^

In this case, the patient had no history of sinusitis or other risk factors and initially presented with neurological symptoms such as fever, headache, and impaired consciousness, leading to a diagnosis of meningitis. Initial brain MRI scans from the outside hospital did not show any pathological changes. The patient developed lower limb weakness that were inconsistent with fever after admission, and COVID-19 was detected during the examination. PET/CT showed interstitial pneumonia with mild FDG elevation and low SUV value, tuberculosis and connective tissue diseases are excluded. The patient was adjusted to include ceftriaxone and vancomycin, according to literature-reported common pathogens of bacterial meningitis.^[8,9]^ Subsequently, the patient’s hypopituitarism and polyuria were noted. A review of the MRI scans revealed infectious changes in the pituitary gland, and further imaging confirmed a pituitary abscess.

Further examination revealed polyneuropathy, monoclonal gammopathy (lamda chain, M-protein), which cannot be explained by pituitary abscess. No monoclonal plasma cells were found in the bone marrow examination. Chronic inflammatory demyelinating polyneuropathy (CIDP), multiple myeloma (MM), and monoclonal gammopathy of undetermined significance (MGUS) were excluded, due to the fact that lymphadenopathy, skin pigmentation, monoclonal gammopathy, polyneuropathy, and endocrinopathies coexisted.

The mandatory diagnostic criteria of POEMS syndrome include polyneuropathy and monoclonal plasma proliferative disorder. The main diagnostic criteria include Castleman disease, sclerotic bone lesions, and elevated VEGF levels. Six minor features include organomegaly, endocrine disorder, skin changes, papilledema, extravascular volume overload, and thrombocytosis. The diagnosis of POEMS syndrome requires mandatory criteria, and at least one major and one minor criterion.^[7]^ The cause of POEMS syndrome remain unclear, but proinflammatory cytokines, seems to be a major pathogenesis. Elevated serum VEGF have been implicated as a major pathophysiologic feature of POEMS syndrome, found to be correlated with the disease activity.^[16]^ However, the average time to the diagnosis of POEMS syndrome is 15 months,^[17]^ the relationship between serum VEGF elevation and polyneuropathy in POEMS syndrome remains controversial, and there were atypical cases with polyneuropathy and M-protein were misdiagnosed due to normal serum VEGF levels.^[16–18]^ Furthermore, Arimura et al showed that serum VEGF levels are related to the clinical features of POEMS syndrome.^[19]^ Sometimes, serum VEGF levels are normal in the early stages of POEMS syndrome. Therefore, it is challenging to diagnose POEMS syndrome in its early stage. It is necessary to combine the clinical characteristics of patients and repeatedly monitor VEGF levels.^[16]^ Regarding this patient, it took about one month to highly suspect the diagnosis of POEMS syndrome, even though the patient’s VEGF levels were normal, which may be attributed to the early stage of POEMS syndrome.

There is no standard treatment for POEMS syndrome. Radiation, corticosteroids, chemotherapy and peripheral blood stem cell transplantation have been used to treat systemic diseases.^[17]^ A longitudinal cohort study conducted by Keddie et al of 100 patients with POEMS syndrome revealed 12 of 100 patients were died, the main causes of death due to POEMS syndrome and cardiopulmonary failure, with 5- and 10-year survival of 90% and 82%.^[17]^ Considering POEMS syndrome may be in a nonprogressive stage due to the normal VEGF levels. In light of the uncertainty regarding the prognosis of POEMS syndrome in this case, lymph node biopsy was delayed, although the patient’s enlarged lymph nodes with increased FDG uptake (SUV max, 11.4). Therefore, there was no clear evidence of Castleman disease and POEMS.

Given the pituitary structural abnormalities, prompt surgical intervention followed by postoperative anti-inflammatory treatment is often preferred in the management of pituitary abscesses.^[10]^ It is generally believed that routine antibiotic treatment for a minimum of 6 weeks postoperatively can reduce instances of abscess recurrence.^[11,14,20]^ In our patient, cerebrospinal fluid and pus cultures were negative, possibly due to prior antibiotic treatment. Symptoms such as fever and headache improved, after treatment with a combination of hormone replacement therapy, endoscopic endonasal transsphenoidal surgery, and antibiotic therapy. The patient was then lost to follow-up after surgery and the antibiotic course was insufficient, which led to recurrence of pituitary abscess. The main symptoms were recurrent fever and headache, which could be relieved after intermittent treatment. The patient’s recent discovery of ascites also further confirmed the diagnosis of POEMS. Unfortunately, the patient did not follow up on VEGF levels, a definitive diagnosis of POEMS could not be supported. And hormonal replacement therapy may be necessary due to potential long-term disruptions in pituitary function. This observation aligns with the findings of Liu et al., who reported that hypopituitarism accompanying pituitary abscess is challenging to treat and often requires lifelong hormone replacement therapy.^[21]^

## 4. Conclusion

Elevated serum VEGF, while being one of the main diagnostic criteria for POEMS syndrome, is normal in a considerable amount of patients that otherwise demonstrate atypical symptoms and features of complex comorbidities. Here, we reported a case of pituitary abscess with high suspicion of POEMS syndrome despite normal VEGF levels. This case emphasizes the critical importance of heightened clinical awareness and comprehensive diagnostic assessments to identify complex comorbidities. It may be reasonable to expand the diagnostic criteria of POEMS syndrome if further atypical cases are reported in the future.

## Acknowledgments

Signed informed consent was obtained from the patient. We are very grateful to the patient’s family for generously authorizing us to share his rare case.

## Author contributions

**Data curation:** Xiangjin Hu.

**Visualization:** Xiangjin Hu.

**Writing – original draft:** Xiangjin Hu.

**Writing – review & editing:** Xiangjin Hu, Li Fang.

**Conceptualization:** Heng Wu, Li Fang.

**Investigation:** Heng Wu.
